# Increased levels of alveolar and airway exhaled nitric oxide in runners

**DOI:** 10.1080/03009734.2017.1317886

**Published:** 2017-05-08

**Authors:** Alexandra Thornadtsson, Nikola Drca, Fabio Ricciardolo, Marieann Högman

**Affiliations:** a Department of Medical Sciences, Respiratory, Allergy and Sleep Research, Uppsala University, Uppsala, Sweden;; b Centre for Research and Development, Uppsala University/Region Gävleborg, Sweden;; c Department of Medicine, Huddinge, Karolinska Institute, Sweden;; d Department of Cardiology, Karolinska University Hospital, Stockholm, Sweden;; e Division of Respiratory Disease, Department of Clinical and Biological Sciences, University of Torino, Turin, Italy

**Keywords:** Athletes, biomarkers, exhaled nitric oxide, pulmonary gas exchange

## Abstract

**Aim:**

The objective of this study was to apply extended NO analysis for measurements of NO dynamics in the lung, divided into alveolar and airway contribution, in amateur runners and marathoners.

**Methods:**

The athletes participated in either a marathon or a half marathon. The athletes self-reported their age, weight, height, training distance per week, competing distance, cardio-pulmonary health, atopic status, and use of tobacco. Measurements of exhaled NO (F_E_NO) with estimation of alveolar NO (C_A_NO) and airway flux (J_aw_NO), ventilation, pulse oximetry, and peak flow were performed before, immediately after, and 1 hour after completing the race.

**Results:**

At baseline the alveolar NO was higher in amateur runners, 2.9 ± 1.1 ppb (*p* = 0.041), and marathoners, 3.6 ± 1.9 ppb (*p* = 0.002), than in control subjects, 1.4 ± 0.5 ppb. J_aw_NO was higher in marathoners, 0.90 ± 0.02 nL s^−1^ (*p* = 0.044), compared with controls, 0.36 ± 0.02 nL s^−1^, whereas the increase in amateur runners, 0.56 ± 0.02 nL s^−1^, did not attain statistical significance (*p* = 0.165). Immediately after the race there was a decrease in F_E_NO in both amateur runners and marathoners, whereas C_A_NO and J_aw_NO were decreased in marathoners only.

**Conclusion:**

Our results support the view that there is an adaptation of the lung to exercise. Thus strenuous exercise increased both airway and alveolar NO, and this might in turn facilitate oxygen uptake.

## Introduction

Nitric oxide (NO) is a gaseous signalling molecule with a variety of biological actions in the human body. It is an important mediator of homeostasis by regulating vascular tone, platelet aggregation, bronchomotor tone, and neurotransmission (1). Further, NO is involved in eradication of pathogens and the inflammatory component of the immune response (2). NO is generated endogenously by conversion of L-arginine to L-citrulline by enzymes of the nitric oxide synthase (NOS) family. At least three isoforms of NOS exist: endothelial NOS (eNOS), neuronal NOS (nNOS), and inducible NOS (iNOS) (1). NO is present in the exhaled breath of humans (3), and it is often used as marker of airway inflammation (4).

During prolonged, repetitive, and intense physical activity the body undergoes adaptations to meet the increased metabolic demand. The appropriate cardiovascular and respiratory responses to exercise are fundamental for adequate oxygen supply. The muscle, cardiovascular, and hematologic systems adapt both structurally and functionally to increase the maximal aerobic capacity (5). It is widely accepted that the pulmonary system does not respond to prolonged aerobic training with structural changes. The functional changes, however, include increased respiratory muscle endurance and strength, as well as increased oxygen uptake (6).

**Figure 1.**
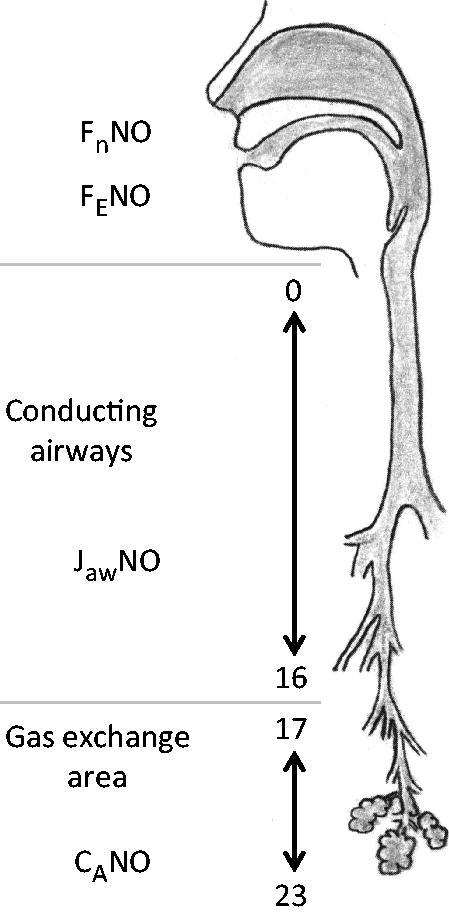
A simplified model of the NO production of the lung. From the gas exchange area (airway generation 23-17) the alveolar nitric oxide (C_A_NO) moves through the conducting airways (generation 16-0) during exhalation. C_A_NO merges with the NO production of the airways (J_aw_NO), and together they are exhaled through the mouth as F_E_NO or through the nose as F_n_NO.

The effect of exercise on fraction of exhaled NO (F_E_NO) has been widely investigated, but there has been no consensus. This is unsurprising as there are significant differences in methodology between the studies. The majority of studies demonstrate decreased F_E_NO after exercise, but a minority demonstrate increased F_E_NO with focus on cardiovascular effects (7). The NO dynamics of the lung, the alveolar and airway contribution to the exhaled NO (Figure 1), can be estimated by an extended NO analysis (8). This has been broadly applied in a variety of lung diseases. The aim of this study was to explore the changes in NO dynamics in amateur runners, marathoners, and healthy subjects that expose themselves to strenuous exercise, with the new guidelines for extended NO analysis (9).

## Materials and methods

In June 1997 and in September 1998 an explorative field study was done where F_E_NO was measured. F_E_NO was measured at three exhalation flows that are suitable for modelling NO dynamics with the linear model of Tsoukias and George (8). The flow dependence of F_E_NO had just been discovered (10,11), and there was no consensus on what exact exhalation flow should be used; later set at 50 mL s^−1^ (12).

### Study subjects

Twenty participants in a half marathon or a full marathon race were enrolled. They were divided into two groups based on their weekly running distance: under 50 km (*n* = 10) referred to as amateur runners, and above 50 km (*n* = 10) referred to as marathoners. The study protocol was explained to the subjects. The study subjects self-reported their age, weight, height, training distance per week, competing distance, cardio-pulmonary health, atopic status, and use of tobacco. The subjects were examined and followed up in a location close to the finish line at the Department of Physiology III, Karolinska Institute, Stockholm, Sweden. The same methodology of NO analysis has been applied in a previous study (13) approved by The Human Ethics Committee at Uppsala University. Sedentary healthy subjects (*n* = 10) from that study were used as controls.

### Exhaled NO analysis

NO measurements were performed in accordance with the American Thoracic Society/European Respiratory Society (ATS/ERS) recommendations (12) but without the vital capacity manoeuvre since a deep breath has been found to be sufficient when performing the measurement (14). Exhaled NO was measured using a NOA 280 (Sievers, Red Wing, MN), and flow rates assessed by a D-lite™ (Datex-Ohmeda, Finland). The system was calibrated using a mixture of 469 ppb NO in nitrogen (AGA, Sweden), and the zero was set by feeding synthetic air (AGA) into a 2-L canister filled with Purafil II chemisorbent with purakol (Lindair, Sweden). The flow sensor was calibrated in the range 0–0.6 L s^−1^ (Dry Cal DC-2 flow calibrator; BIOS International, Butler, NJ). Validations of the calibration and flow rate of the sampling system were made on a daily basis, and the zero was ensured before each measurement. The expiratory pressure for all subjects ranged between 5 cm H_2_O and 20 cm H_2_O in order to exclude a NO contribution from the nasal cavity. Exhaled NO from the mouth was measured at three flows above 0.1 L s^−1^ (0.1 L s^−1^, 0.2 L s^−1^, and 0.3 L s^−1^) and abbreviated F_E_NO_0.1_ for 0.1 L s^−1^. Exhalation from the nose was measured at 0.1 L s^−1^ (F_n_NO_0.1_). The mean values of three breaths (or two, if the NO concentrations were identical of the two breaths) of each flow rate were used for statistical analysis.

The linear model of Tsoukias and George (8) was applied and is a first-order approximation with a simple linear regression estimated via ordinary least squares. The model uses three flow rates above 0.1 L s^−1^, and mean values of two exhalations at each flow were used (9). The value for the regression line is computed to give the quality of the measurements, and an accepted quality was considered with an *r* value above 0.85. The model gives estimates of NO parameters, i.e. alveolar NO (slope of the line, C_A_NO) and airway flux of NO values (intercept of the *y*-axis, J_aw_NO) from a plot of flow rate and exhaled NO production from the respiratory system.

### Ventilation, pulse oximetry, and peak flow

A CS/3 critical care monitor (Datex-Ohmeda, Instrumentarium Corp., Helsinki, Finland) was used to measure minute ventilation, oxygen consumption (VO_2_), peripheral oxygen saturation (SpO_2_), end-tidal CO_2_ (ETCO_2_), respiratory rate (resp rate), and heart rate (HR). A Mini-Wright standard peak flow meter (Clement Clark International, Harlow, UK) was used to identify airway obstruction after running.

### Protocol for the athletes

Baseline measures were performed in a sitting position 2 hours prior to the race. Exhaled NO was measured at different flow rates, followed by ventilation, and lastly the peak expiratory flow was measured. The measurements were repeated immediately after, i.e. within 5 min, and 1 hour after the race. All study-participating athletes completed their race. Data from this study were collected in 1997–1998 as part of a student project. At that time point no ethical applications were required for that type of studies. These 20-year-old data have now been re-used and a new NO modelling applied. While considering potential ethical shortcomings we still find it likely that calculations and interpretations of such modelling might be of interest to the scientific community. According to Swedish regulations ethical applications in retrospect cannot be considered.

### Statistics

The normal distribution of the data was tested with the Kolmogorov–Smirnov test. NO parameters that could not be proven to have a normal distribution were treated as non-parametric (F_E_NO and J_aw_NO). Correlations were tested with Spearman rank order correlation. Differences between the groups were tested with Kruskal–Wallis test and Mann–Whitney *U* test. Differences within the groups were tested with Friedman ANOVA and Wilcoxon signed rank test. SPSS v. 21 for Windows (SPSS Inc., Chicago, IL) was used for all statistical calculations. Data have been presented as arithmetic mean and quartiles and as geometrical mean and quartiles for F_E_NO_0.1_ and J_aw_NO. A *p* value of <0.05 was regarded as significant.

## Results

None of the study subjects reported any history of cardio-pulmonary disease. They were all non-smokers. One amateur runner and one marathoner reported a history of pollen allergies; however, they did not experience any symptoms on the day of the race. Four amateur runners and eight marathoners performed a half marathon race, and six amateur runners and two marathoners performed a marathon race.

The quality of the NO modelling was tested by plotting flow rate and NO production for each subject and computing a regression line. The value for the regression line was 0.94 (0.88, 0.99) for control subjects, 0.97 (0.98, 1.0) for amateur runners, and 0.97 (0.94, 0.99) for marathoners.

### Baseline

**Figure 2.**
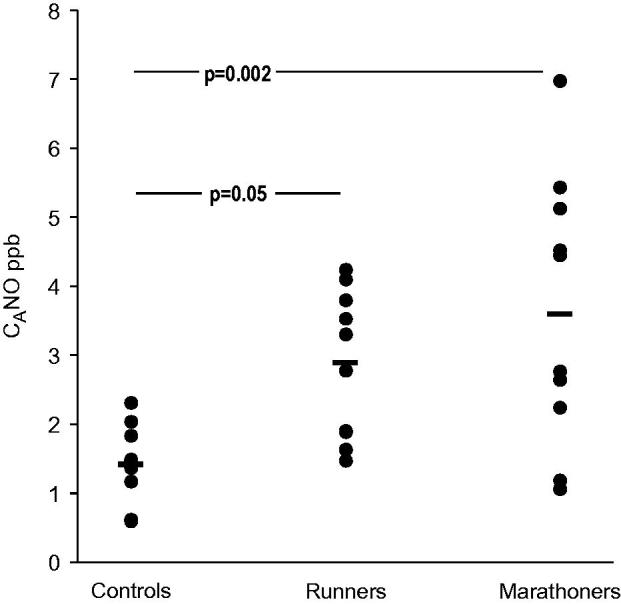
Alveolar nitric oxide (C_A_NO) in controls, amateur runners, and marathoners.

The marathoners were younger, taller, and had a lower BMI compared with control subjects (Table 1). F_E_NO_0.1_ was higher in runners (*p* = 0.015) and marathoners (*p* < 0.001) compared with controls, but J_aw_NO was higher in marathoners only (*p* = 0.04). There was an increase in C_A_NO when comparing controls and amateur runners and controls and marathoners (Figure 2).

**Table 1.** Descriptive statistics for controls, amateur runners, and marathoners.

	Controls	Runners	Marathoners	*p* Value
M/F	*n* = 3/7	*n* = 9/1	*n* = 10/0	
Age (M/F)	41 (32, 49)	31 (23, 39)	29 (25, 33)^b^	0.049
Height, cm	169 (161, 176)	178 (172, 184)	183 (179, 187)^b^	0.012
Weight, kg	67 (58, 75)	73 (63, 79)	71 (66, 78)	0.541
BMI	23 (22, 24)	23 (21, 25)	21 (21, 22)^b^	0.045
F_E_NO_0.1_, ppb^a^	4.8 (4.0, 6.1)	8.4 (5.6, 9.8)^b^	12.4 (8.2, 21.4)^b^	0.002
J_aw_NO, nL s^−1^^a^	0.36 (0.25, 0.60)	0.56 (0.28, 0.91)	0.90 (0.43, 1.91)^b^	0.051
C_A_NO, ppb	1.4 (1.0, 1.9)	2.9 (1.8, 3.9)^b^	3.6 (2.0, 5.2)^b^	0.005

Data are given as means (quartile 25, 75) and for data with skewed distribution geometrical mean (quartile 25, 75).

a Geometrical mean; Kruskal–Wallis.

b Mann–Whitney *U* test.

**Figure 3.**
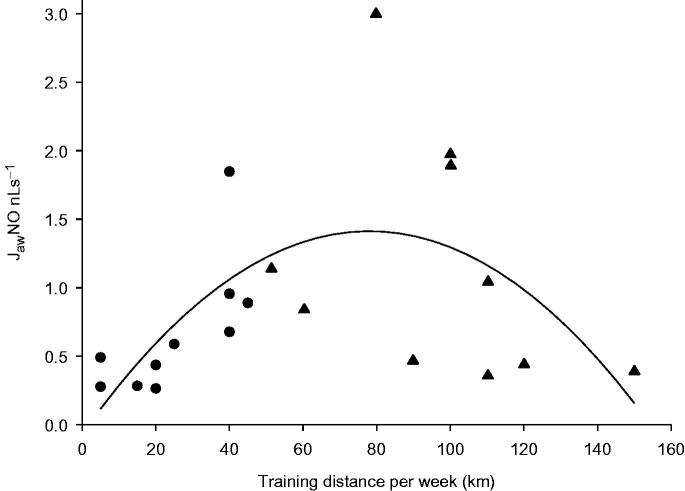
The weekly training distance reported by runners (•) and marathoners (▴). A quadratic regression line was fitted to all athletes, and the adjusted *r^2^* was 0.28, *p* = 0.013.

The training distances per week were higher in marathoners, 97 (75, 112) km, as compared with those of amateur runners, 26 (12, 40) km (*p* < 0.001). In amateur runners there was a correlation between J_aw_NO and training distance per week, *r* = .78, *p* = 0.007. In marathoners this correlation was negative, *r* = –.57, and failed to reach statistical significance (*p* = 0.087). A quadratic regression line was fitted to all athletes, and the adjusted *r^2^* was .28. The *p* value for the regression coefficient was 0.008 for the linear term of the model and .013 for the quadratic term (Figure 3).

### After the race

Immediately after the race in amateur runners there was an increase in HR (*p* = 0.005) and a decrease in F_E_NO_0.1_ (*p* = 0.022) in comparison with before the race. One hour later the HR was still increased (*p* = 0.005). In marathoners the HR was increased (*p* = 0.005), and ETCO_2_ (*p* = 0.005) and F_E_NO_0.1_ (*p* = 0.005) decreased immediately after the race and 1 hour later (*p* = 0.022, *p* = 0.007, and *p* = 0.005). J_aw_NO (*p* = 0.022) and C_A_NO (*p* = 0.037) decreased immediately after the race in marathoners (Table 2).

**Table 2.** Results of lung function, heart rate, and NO parameters before, immediately after, and 1 hour after a race by amateur runners and marathoners.

	Amateur runners				Marathoners			
	Before	After	1 Hour after	*p* Value	Before	After	1 Hour after	*p* Value
HR, bpm	72 (64, 78)	99 (77, 113)^a^	91 (83, 98)^a^	<0.001	67 (52, 82)	88 (77, 98)^a^	76 (66, 86)^a^	<0.001
PEF, L min^−1^	646 (585, 722)	623 (575, 683)	638 (575, 712)	0.254	646 (618, 685)	637 (595, 695)	632 (593, 680)^a^	0.044
SpO_2_, %	99 (98, 99)	98 (98, 99)	98 (97, 99)	0.113	99 (98, 99)	97 (97, 98)	98 (97, 99)	0.107
ETCO_2_, %	5.6 (5.1, 5.9)	5.3 (5.0, 5.8)	5.4 (5.1, 5.7)	0.196	5.8 (5.6, 6.2)	5.0 (4.1, 5.8)^a^	5.3 (5.0, 5.8)^a^	<0.001
F_E_NO_0.1_, ppb^b^	8.4 (5.6, 9.8)	5.5 (3.1, 8.4)^a^	6.6 (5.2, 10.4)	0.045	12.4 (8.2, 21.4)	6.6 (4.2, 13.8)^a^	7.3 (4.4, 16.0)^a^	<0.001
F_n_NO_0.1_, ppb^b^	32 (25, 36)	21 (17, 29)^a^	25 (22, 32)^a^	0.008	23 (18, 25)	21 (16, 25)	20 (14, 29)	0.497
J_aw_NO, nL s^−1^^b^	0.56 (0.28, 0.91)	0.40 (0.20, 0.64)	0.45 (0.35, 0.89)	0.067	0.90 (0.43, 1.91)	0.60 (0.34, 1.14)^a^	0.68 (0.38, 1.50)	0.045
C_A_NO, ppb	2.9 (1.8, 3.9)	2.0 (1.5, 2.7)	2.5 (1.8, 3.2)	0.273	3.6 (2.0, 5.2)	2.1 (0.9, 3.8)^a^	1.7 (0.4, 2.5)	0.061

Data are given as means (quartile 25, 75) and for data with skewed distribution geometrical mean (quartile 25, 75). Friedman ANOVA.

a Wilcoxon matched pairs test.

b Geometrical mean.

**Figure 4.**
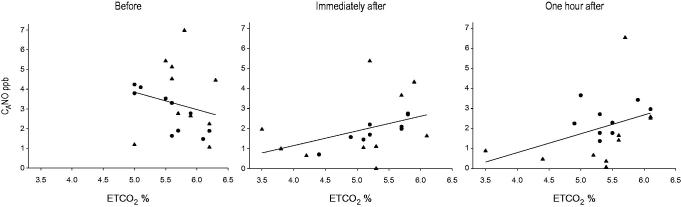
ETCO_2_ and alveolar nitric oxide (C_A_NO) before, immediately after, and 1 hour after the race, in runners (•) and marathoners (▴). Before the race the correlation was: *r* = –0.30, *p* = 0.199; immediately after: *r* = 0.56, *p* = 0.013; and 1 hour after: *r* = 0.45, *p* = 0.052 for all runners.

In amateur runners there was a correlation between C_A_NO and ETCO_2_ before the race, *r* = –0.85, *p* = 0.002, and immediately after the race, *r* = .903, *p* = 0.001 (Figure 4). However, 1 hour after the race there was no correlation. In marathoners there were no correlations between C_A_NO and ETCO_2_ before and immediately after the race. However, 1 hour after the race the correlation was *r* = .734, *p* = 0.016.

Nasal NO before the race was within normal values for both amateur runners and marathoners. However, marathoners had a lower F_n_NO_0.1_ value before the race (*p* = 0.041). In amateur runners there was a decrease in nasal NO immediately (*p* = 0.011) and 1 hour after the race (*p* = 0.028). The race had no impact on nasal NO in marathoners (Table 2).

Minute ventilation, oxygen consumption (VO_2_), and respiratory rate were measured only in the four runners and six marathoners who participated in the half marathon (Table 3). For all athletes the minute ventilation was increased both immediately after (*p* = 0.017), and 1 hour after the race (*p* = 0.007), while oxygen uptake was only increased immediately after the race (*p* = 0.019). Respiratory rates were decreased 1 hour after the race (*p* = 0.016).

**Table 3.** Results of ventilation and respiratory rate before, immediately after, and 1 hour after a race by amateur runners (*n* = 4) and marathoners (*n* = 6).

	Before	After	1 Hour after	*p* Value
Ventilation, L min^−1^	8.4 (5.7, 11.0)	10.9 (7.5, 14.2)^a^	10.2 (7.7, 11.8)^a^	0.006
VO_2_, mL min^−1^	271 (205, 328)	367 (295, 415)^a^	322 (225, 395)	0.067
Resp rate, bpm	13 (12, 14)	12 (9, 13)	11 (9, 13)^a^	0.032

Data are given as mean (quartile 25, 75). Friedman ANOVA.

a Wilcoxon matched pairs.

## Discussion

This study examined the NO dynamics in the lung of runners before and after a race. C_A_NO and F_E_NO_0.1_ were higher in amateur runners and marathoners before the race. J_aw_NO showed a strong positive correlation to training distance in the runners but showed a negative trend in the marathoners. The running performance had a greater effect on the NO parameters in marathoners, in that C_A_NO and J_aw_NO levels were decreased compared with the runners.

There is little evidence that the lung adapts to strenuous exercise (6). However, increased diffusing capacity of the lung has been observed in swimmers that could be explained by larger lung volumes. Swimmers exhale under water, a situation that creates a positive exhalation pressure that leads to extension of the lung (15). There was a higher C_A_NO in the athletes at baseline. Increased C_A_NO has also been found in obstructive sleep apnoea syndrome (16), severe asthma (17), and chronic obstructive pulmonary disease (18). The common feature for all these diseases is the presence of various degrees of hypoxaemia. The increased C_A_NO in our athletes can possibly be explained by repetitive exposure to hypoxaemia during the heavy training period preparing for the race.

In athletes performing strenuous exercise there is a risk of hypoxaemia and lung oedema, and highly trained athletes are at risk to develop hypoxaemia due to heavy exercise (19–21). When the hypoxaemia is prolonged, oedema formation may occur. This has been found to be evident in highly trained athletes (22). Zavorsky et al. reported mild interstitial pulmonary oedema in 20% of healthy subjects after participation in a marathon (23). In high-altitude pulmonary oedema (HAPE), there is decreased blood oxygenation, and subjects who produce sufficient amounts of NO have been found to resist HAPE (24). Nitric oxide from the endothelium prevents capillary leakage, which in turn could prevent pulmonary oedema (25). Lower exhaled NO values have been observed in HAPE-prone subjects compared with controls (24). NO is oxygen-dependent, and there is an optimal balance between oxygen tension and the production of NO (26). It has been widely known for long that *inhaled* NO enhances oxygen uptake due to improvement in ventilation/perfusion matching of the lung (27). Thus the pulmonary vessels in the well-ventilated areas of the lung dilate, thereby increasing the surface area for gas exchange. In endurance-trained athletes the pulmonary capillary blood volume has been found to be increased (28). In our study, 8 of 20 athletes had decreased levels of C_A_NO (by more than 50%) after their race. Based on these results, we hypothesized that the oedema could be formed due to a decrease in alveolar NO, but we did not assess oedema formation.

Endurance-trained athletes have an increased maximum oxygen uptake (VO_2_-max) as well. One limiting factor for VO_2_-max is the pulmonary diffusing capacity, which is more evident in elite-trained athletes due to an increased cardiac output with a subsequent decrease in red blood cell transit time. Increased surface area of gas exchange could possibly decrease this limiting factor. This could be done by either angiogenesis of the pulmonary system and/or increased C_A_NO with dilation of the pulmonary capillary bed and subsequent increase in ventilation/perfusion matching. Our data concerning the correlation of ETCO_2_ to C_A_NO might suggest that NO is involved in oxygen diffusion and ventilation.

The role of NO in the airway epithelia has been extensively investigated. NO is important in the defence system, since it has anti-microbial, anti-inflammatory, and anti-oxidative properties, and also by regulating the ciliary function of the respiratory epithelium that mechanically transports inhaled particles (1). In our athletes there was a trend towards higher airway NO flux with increased training distance per week, but when the distance exceeded 100 km the airway NO flux decreases towards control values. The correlation between the airway NO flux and training distance per week is positive and linear up to 60 km per week. Then there is a greater variation, probably due to individual factors. One possible explanation to this is that different individuals react differently to an increased endurance training. Nieman et al. investigated infectious episodes in runners before and after a marathon race and found a higher odds ratio for infection with a training distance per week above 97 km (29). The increased infection risk can possibly be explained by a decrease in airway NO. Our runners showed a reduction in exhaled NO when the training distance exceeded 100 km per week. It might well be that the protective effect of NO on the airway induced by training may be lost or the subjects who had the highest weekly training distance might be at risk of overreaching or more severely condition such as overtraining. Overtraining syndrome (OTS) is among other things characterized by increased susceptibility to inflammation following episodes of repetitive strenuous exercise. Several hypotheses have been proposed. One hypothesis explaining OTS is increased oxidative stress (30). Oxidative stress is known to reduce NO by formation of potent oxidizing reactive nitrogen species, such as peroxynitrite, which is able to damage proteins, lipids, and DNA, leading to impaired cellular functions (31). One other explanation to the decrease in NO flux after a 100-km training distance per week is that high-level exercise may cause injury to the airway epithelium due to severe hyperpnoea and subsequent dehydration, with increased mechanical forces exerted on airway surfaces reducing airway NO diffusion (32).

Intense and prolonged exercise is associated with increased risk for upper respiratory tract infection due to alterations in the immune system (33). NO is known to be a regulator of ciliary beat frequency by enhancing the motility of cilia (34). One study found a decrease in mucociliary clearance measured by nasal mucociliary transit time in long-distance runners at recreational level compared with healthy sedentary controls (35). In our study nasal NO was within normal values (14) although lower at baseline in marathoners compared with the amateur runners. There was a decrease in nasal NO in amateur runners after the race but not in marathoners. When the mucociliary transit time was measured there was an increase in transit time after the marathon race (35). This increase in mucociliary transit time could possibly be explained by the decrease in nasal NO that we found in our study.

We conclude that our finding of increased alveolar NO in amateur runners and marathoners suggests that there is a potential for the lung to adapt to strenuous exercise. Such a capacity change of the lung might be accompanied by an enhanced oxygen uptake due to the vascular effects of NO that in turn improve the perfusion of the alveoli. This hypothesis needs further investigation in other endurance athletes such as elite bicycle riders and skiers.
